# OUTCOMES OF DISCONTINUING CHOLINESTERASE INHIBITORS IN NURSING HOME RESIDENTS WITH SEVERE DEMENTIA

**DOI:** 10.1093/geroni/igz038.2681

**Published:** 2019-11-08

**Authors:** Joshua D Niznik, Xinhua Zhao, Meiqi He, Sherrie Aspinall, Joseph Hanlon, David Nace, Joshua Thorpe, Carolyn Thorpe

**Affiliations:** 1 University of Pittsburgh, Pittsburgh, Pennsylvania, United States; 2 University of North Carolina Eshelman School of Pharmacy, Chapel Hill, North Carolina, United States

## Abstract

Some clinical guidelines advocate for the withdrawal of cholinesterase inhibitors (ChEIs) in patients with severe dementia. However, there have been no studies of the outcomes of deprescribing ChEIs specifically in patients with severe dementia, and concerns about subsequent worsening of behavioral symptoms may serve as a barrier to ChEI discontinuation. Our objective was to evaluate the impact of deprescribing ChEIs on aggressive behaviors and depression severity in older nursing home (NH) residents with severe dementia. We conducted a retrospective cohort study using Medicare claims, Part D prescriptions, Minimum Data Set (MDS) v3.0, Area Health Resource File, and Nursing Home Compare, for non-skilled NH residents aged 65+ with severe dementia receiving AChEIs with ≥2 MDS assessments in 2016 (n=30,788). The Aggressive Behavior Scale (ABS) and the Patient Health Questionnaire (PHQ-9) evaluated aggression and depression, respectively. Marginal structural models with inverse probability of treatment weights evaluated the association of deprescribing with outcomes, accounting for time-dependent confounding. The sample was primarily white (78.7%), female (76.6%), >80 years old (77.6%), and 22.8% were deprescribed ChEIs. In adjusted models, deprescribing was not associated with aggression (0.002 point increase in ABS, p=0.90) or depression (0.04 point increase in PHQ-9, p=0.50). Deprescribing ChEIs in NH residents with severe dementia did not lead to an increase in aggressive behaviors or depression severity. Our findings provide insight into the potential risks and benefits associated with deprescribing ChEIs and help inform decision-making in patients with severe dementia.

